# The effect of nicotine-containing products and fetal sex on placenta-associated circulating midpregnancy biomarkers

**DOI:** 10.1186/s13293-022-00443-1

**Published:** 2022-07-15

**Authors:** Birgitte Kordt Sundet, Ina Kreyberg, Anne Cathrine Staff, Karin Cecilie Lødrup Carlsen, Karen Eline Stensby Bains, Jens Petter Berg, Berit Granum, Guttorm Haugen, Gunilla Hedlin, Christine Monceyron Jonassen, Live Solveig Nordhagen, Björn Nordlund, Eva Maria Rehbinder, Knut Rudi, Corina Silvia Rueegg, Katrine Dønvold Sjøborg, Håvard Ove Skjerven, Cilla Söderhäll, Riyas Vettukattil, Meryam Sugulle

**Affiliations:** 1 Faculty of Medicine, Institute of Clinical Medicine, University of Oslo, Oslo, Norway; 2 Division of Obstetrics and Gynaecology, Oslo University Hospital, Nydalen, Postbox 4956, 0424 Oslo, Norway; 3 Division of Paediatric and Adolescent Medicine, Oslo University Hospital, Oslo, Norway; 4 Department of Medical Biochemistry, Oslo University Hospital, Oslo, Norway; 5 Department of Environmental Health, Norwegian Institute of Public Health, Oslo, Norway; 6 Astrid Lindgren Children’s Hospital, Karolinska University Hospital, Stockholm, Sweden; 7 Department of Women’s and Children’s Health, Karolinska Institutet, Stockholm, Sweden; 8 Faculty of Chemistry, Biotechnology and Food Science, Norwegian University of Life Sciences, Ås, Norway; 9 Genetic Unit, Centre for Laboratory Medicine, Østfold Hospital Trust, Kalnes, Norway; 10 VID Specialized University, Oslo, Norway; 11 Department of Dermatology, Oslo University Hospital, Oslo, Norway; 12 Oslo Centre for Biostatistics and Epidemiology, Oslo University Hospital, Oslo, Norway; 13 Department of Obstetrics and Gynecology, Østfold Hospital Trust, Kalnes, Norway

**Keywords:** Angiogenic proteins, Placenta, Moist tobacco, Nicotine, Fetal sex, PreventADALL

## Abstract

**Background:**

In utero exposure to nicotine, largely assessed by smoking, is a risk factor for impaired offspring health, while potential effects of non-combustible nicotine use such as snus (oral moist tobacco), are less well-known. Maternal serum concentrations of placental growth factor (PlGF) and soluble fms-like tyrosine kinase-1 (sFlt-1) may be viewed as “placenta health markers”, known to differ by fetal sex. Maternal smoking during pregnancy has been associated with lower levels of circulating sFlt-1, while the effect of snus on placenta-associated angiogenic factors is unknown. Our aim was to explore if snus and/or smoking exposure was associated with midpregnancy maternal levels of sFlt-1, PlGF and sFlt-1/PlGF ratio if these associations were modified by fetal sex.

**Methods:**

Midpregnancy (16–22 gestational weeks) serum from 2603 Scandinavian women enrolled in the population-based multi-center PreventADALL (Preventing Atopic Dermatitis and ALLergies in children) study was analysed for sFlt-1 and PlGF concentrations by electrochemiluminescence, deriving the sFlt-1/PGF ratio. Nicotine use was assessed by electronic questionnaires at enrollment in 2278 of the women. Univariable and multivariable linear regression models on log transformed outcomes were used to assess the association between nicotine use and biomarker levels. Interaction terms were included to identify whether the associations were modified by fetal sex.

**Results:**

Median sFlt-1, PlGF and sFlt-1/PlGF ratios among women with nicotine exposure information were similar to those of all included women and differed by fetal sex. Current snus use was significantly associated with reduced maternal circulating PlGF levels in adjusted analyses [*β* − 0.12, (95% CI − 0.20; 0.00) compared to never use, *p* = 0.020]. A significant interaction between fetal sex and snus exposure was observed for PIGF (*p* = 0.031). Prior or periconceptional snus use was significantly associated with PIGF in male fetus pregnancies [*β* − 0.05 (95% CI − 0.09 to (− 0.02)) and *β* − 0.07 (95% CI − 0.12 to (− 0.02)) compared to never use, *p* = 0.002]. Smoking was not significantly associated with any circulating biomarkers levels.

**Conclusions:**

Midpregnancy maternal angiogenic profile differed by periconceptional snus use and fetal sex. Snus exposure, perceived as “safe” by users, before or during pregnancy seems to affect midpregnancy placental health in a sex dimorphic manner.

**Supplementary Information:**

The online version contains supplementary material available at 10.1186/s13293-022-00443-1.

## Introduction

Maternal circulating proangiogenic placental growth factor (PlGF) and antiangiogenic soluble fms-like tyrosine kinase-1 (sFlt-1) may be seen as “placenta health markers”, since altered levels of these proteins and their ratio are associated with placenta dysfunction syndromes like preeclampsia and fetal growth restriction [1], as well as with other cases of increasing placental cellular (syncytiotrophoblast) stress [2–4].

In utero exposure to nicotine increases the risk of impaired offspring health, while early cessation may attenuate the risk of some adverse outcomes towards the level of non-tobacco users [5–8]. Among Scandinavian women in reproductive age, smoking rates are declining, also in pregnancy, whereas the use of other nicotine products, such as snus (oral moist tobacco) is increasing [9]. Population-based birth registry studies have indicated increased risk of preeclampsia, stillbirth and preterm delivery [8] related to in utero exposure to snus, whereas the effect on birth weight is less clear [5, 10, 11]. The Scandinavian prospective mother–child PreventADALL (Preventing Atopic Dermatitis and Allergies in children) study [12] recently identified snus as the most frequently used tobacco product, reported by 6.9% of women during pregnancy, however mostly restricted to periconceptional use [9]. Current smoking has been associated with lower maternal concentrations of antiangiogenic sFlt-1 [13], probably secondary to placental carbon monoxide effects [14]. Compared to cigarettes, nicotine uptake from snus through the oral mucosa is slower [15]. Nicotine levels reached in serum are higher for cigarettes initially, but snus provides a higher level over time with a prolonged decline resulting in a greater systemic dose [15]. The effect of snus on placentation, placental function and maternal angiogenic biomarker levels is less investigated.

A sexual dimorphism in maternal circulating angiogenic factor levels has previously been found in first trimester [16] as well as throughout pregnancy [17], with female fetus pregnancies being associated with higher sFlt-1 concentrations and higher sFlt-1/PlGF ratio. Whether the effect of nicotine on placenta associated biomarkers differs by fetal sex is not known.

We hypothesize that maternal nicotine use in the form of snus is associated with altered placental health as evaluated by maternal circulating angiogenic biomarker levels. Our aim was to explore if maternal nicotine use was associated with mid-pregnancy maternal levels of sFlt-1, PlGF and sFlt-1/PlGF ratio and if these associations were modified by fetal sex.

## Methods

### Study design

**Fig. 1:**
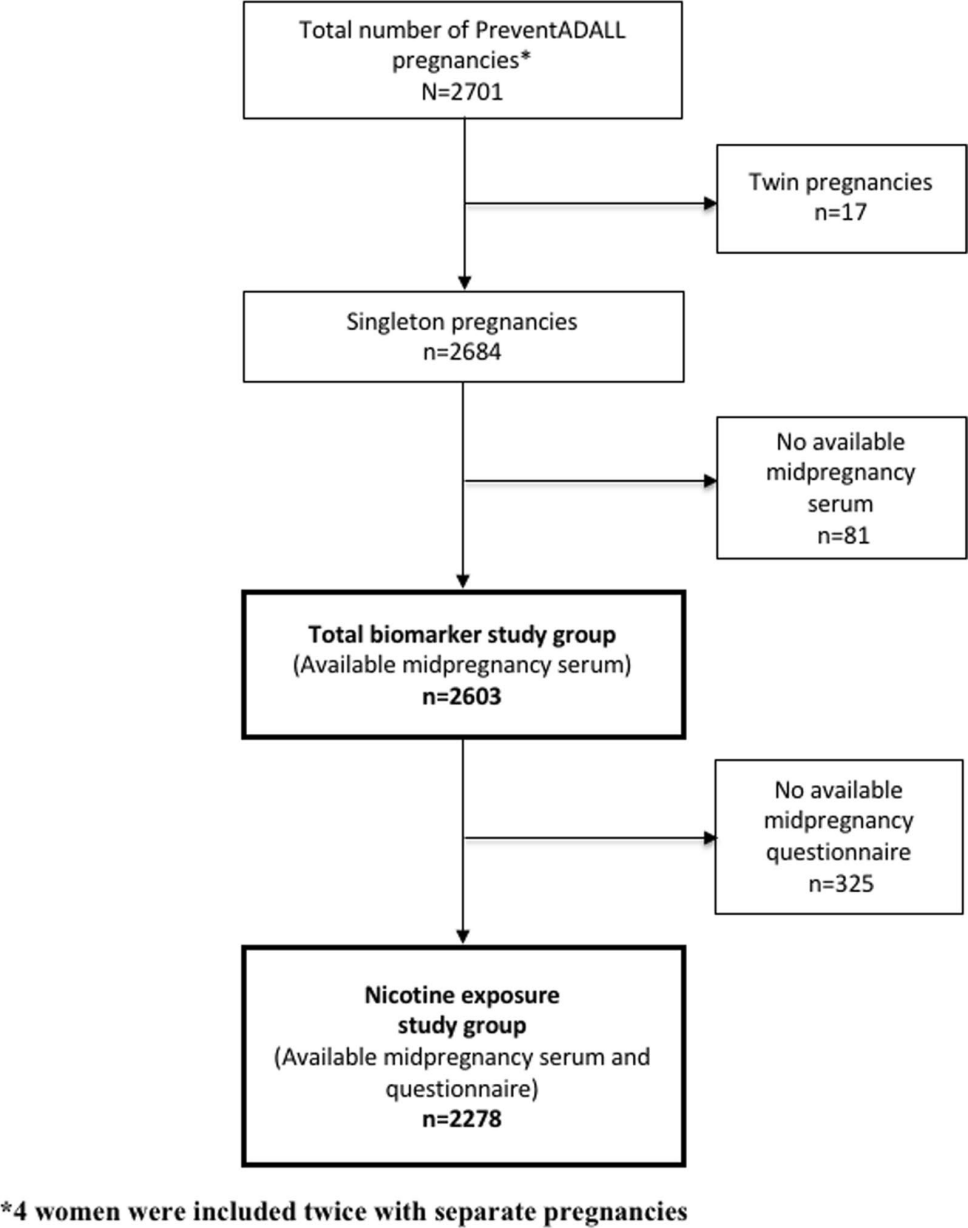
Study enrollment flow chart. *4 women were included twice with separate pregnancies. 5 (0.2%) women reported use of e-cigarettes and/or NRTs at some time in pregnancy (stopped when recognized pregnancy or current use). Of these women, 2 reported smoking cessation when recognized pregnancy and were included in this group. The remaining 3 reported no use of snus or smoking at some time in pregnancy, but reported previous smoking and/ or use of snus and were included in these groups, respectively

The present study is based on the population-based PreventADALL study [9, 11, 12] including all women with available midpregnancy serum samples from singleton pregnancies (*n* = 2603, denoted “Total biomarker study group”, Fig. 1), collected at study enrollment at the national routine ultrasound examination around gestational week (GW) 18. Study enrollment took place from December 2014 through October 2016 at Oslo University Hospital and Østfold Hospital Trust in Norway and Karolinska Institutet in Sweden. Nicotine exposure information was self-reported by electronic questionnaires at study enrollment and available in 2278 of the 2603 women (denoted “Nicotine exposure study group”, Fig. 1). Four women contributed with two separate pregnancies each. Detailed information on the PreventADALL study has been published previously [9, 11, 12, 18].

### Blood sampling and biomarker analysis

At study enrollment around GW 18, blood was drawn from non-fasting women and serum was stored until analysis, as described elsewhere [12]. After thawing, all samples were analyzed at one site (Oslo University Hospital, Dept. of Medical Biochemistry), blinded to clinical data. Maternal serum concentrations of sFlt-1 and PlGF were determined using the fully automated Elecsys® sFlt-1 and Elecsys® PlGF assays on the cobas e801 electrochemiluminescence immunoassay platform (Roche Diagnostics), according to the manufacturer’s instructions. All concentrations were within the detectable ranges of the Elecsys® PlGF and sFlt-1 assays (3–10 000 pg/mL and 10–85 000 pg/mL, respectively). The analytical coefficients of variation were ≤ 2.1% for PlGF and ≤ 1.8% for sFlt-1.

### Outcome measures, nicotine exposure and covariates

The main outcome measures were maternal circulating midpregnancy concentrations of sFlt-1 and PlGF (pg/mL), and the sFlt-1/PlGF ratio.

From an electronic questionnaire at study enrollment information on baseline characteristics were recorded and nicotine exposure was reported by type, frequency, and period of use including before or at the start of pregnancy and current use at the time of enrollment, as published previously [9, 11, 12, 18]. For nicotine exposure analyses women were categorized into users of snus and/or cigarette smoking by the following respective categories; never, stopped before pregnancy, stopped when recognized pregnancy and current use. Throughout the manuscript the term smoking relates to cigarette smoking.

The covariates used included: gestational age at enrollment calculated by fetal femur length measured at routine second trimester ultrasound examination [12, 18], and grouped into ‘16–17’, ‘18’, ‘19–20’ and ‘21–22’ weeks. Maternal age at enrollment was categorised as: ‘ < 30’, ‘30–35’, ‘ > 35–40’ and ‘ > 40’ years based on birth date. Parity was categorized as ‘0’, ‘1’ and ‘ > 1’. Prepregnancy BMI was calculated based on height measured at enrollment and self-reported prepregnancy weight, and categorized as ‘underweight’ (< 18.5 kg/m^2^), ‘normal’ (18.5–24.9 kg/m^2^), ‘overweight’ (25.0–29.9 kg/m^2^) and ‘obese’ (≥ 30.0 kg/m^2^). Fetal sex as categories female and male.

### Statistical analyses

Categorical variables are presented in numbers and percentages, and continuous variables as means ± SD or medians with interquartile ranges as appropriate. We describe angiogenic biomarker levels for the different nicotine exposure groups overall and by fetal sex. Univariable and multivariable linear regression models were used to assess the association between snus use and smoking and angiogenic biomarkers. Nicotine exposure categorized as ‘never’ (snus, and/or smoking) was used as the reference. To isolate the effect of snus use alone at some time in pregnancy, dual users (smoking and snus use) were removed from the snus category and included in the smoking category. Due to non-normality, the biomarkers were log-transformed for all regression analyses. Effect estimates were back translated using the exponential function prior to presentation in text.

Multivariable models were adjusted for the following preselected potential confounders: gestational age, maternal age, prepregnancy BMI, parity and fetal sex all with potential biological effect on the studied biomarkers [17, 19–21]. The model with snus use as main exposure was additionally adjusted for previous smoking history. In a next analyses step, univariable and multivariable linear regression models were used to assess the effect of fetal sex on the angiogenic biomarkers. Fetal sex category ‘female’ was used as the reference. Tests for interaction between fetal sex and nicotine exposure were performed separately for snus use and smoking in the multivariable model, and in case of significant interaction, the respective multivariable model was stratified by fetal sex. The significance level was set to 5%. Four women contributed with two pregnancies each. We performed a sensitivity analysis where only the first pregnancy of each of these four women was included, to assess potential bias because of non-independent pregnancies. All analyses were performed by IBM SPSS statistics version 25 (Chicago, IL, U.S.A.).

## Results

The two presented study groups, i.e., the “Total biomarker study group” consisting of 2603 women and the “Nicotine exposure study group” comprising 2278 (87.5%) of the 2603 women (Fig. 1), were similar with regard to maternal and pregnancy characteristics (Table 1).

**Table 1:** Clinical characteristics for Total biomarker study group (*n* = 2603) and Nicotine exposure study group (*n* = 2278)

Maternal and pregnancy characteristics	Total biomarker study group (*n* = 2603)		Nicotine exposure study group (*n* = 2278)	
	N	%	N	%
*Fetal sex*				
Male	1371	53.0	1201	53.0
Female	1214	47.0	1066	47.0
Gestational age at blood sampling (weeks)*, mean ± SD	19.8 ± 6.2		19.8 ± 6.1	
16–17	199	7.7	168	7.5
18	811	31.5	695	30.8
19–20	993	38.6	859	38.1
21–23	573	22.2	532	23.6
*Maternal age at enrollment (years),* mean ± SD	32.3 ± 4.2		32.4 ± 4.2	
< 30	900	34.5	770	33.8
30–35	1120	43.0	993	43.6
> 35–40	500	19.2	441	19.4
> 40	83	3.2	74	3.2
*Maternal prepregnancy body mass index (kg/m*^*2*^*),* mean ± SD	24.8 ± 3.7		24.8 ± 3.7	
Underweight < 18.5	87	3.4	76	3.4
Normal weight 18.5–24.9	1902	74.9	1669	75.1
Overweight 25.0–29.9	405	16.0	361	16.3
Obese ≥ 30.0	145	5.7	115	5.2
*Parity (previous deliveries)*				
0	1694	65.1	1369	60.1
1	717	27.5	717	31.5
> 1	192	7.4	192	8.4
*Country of origin*				
Norway or Sweden	2020	77.6	2020	88.7
Rest of the world	258	9.9	258	11.3
*Education (years)*				
Preliminary school only (9–10 years)	18	0.7	18	0.8
High school only	230	8.8	230	10.1
Higher education < 4 years	730	28.0	730	32.2
Higher education ≥ 4 years	1289	49.5	1289	56.8
Other	2	0.1	2	0.1
*Snus*				
Never	1762	67.7	1762	77.3
Stopped before pregnancy	343	13.2	343	15.1
Stopped when recognizing pregnancy	160	6.2	160	7.0
Current	13	0.5	13	0.6
*Smoking*				
Never	1762	67.7	1762	77.3
Stopped before pregnancy	404	15.5	404	17.6
Stopped when recognizing pregnancy	97	3.7	97	4.3
Current	15	0.6	15	0.7

N, number; SD, Standard Deviation; %, percentage

In the nicotine exposure study group, the number of women who stopped using nicotine products when they recognized pregnancy was 160 (7.0%) for snus use and 97 (4.3%) for smoking, whereas current use at 18 weeks gestational age was 13 (0.5%) for snus use and 15 (0.7%) for smoking (Table 1). All 11 women who reported dual use of cigarettes and snus, quit when recognized pregnancy and none reported current dual use at 18 GW.

The median sFlt-1, PlGF and sFlt-1/PlGF ratios were similar in the “Total biomarker study group” and the “Nicotine exposure group” (Table 2). Among the 2603 women, the median maternal sFlt-1 concentration was 1258.0 pg/mL (IQR 938.0–1754.0), median PlGF was 192.0 pg/mL (IQR 142.0–260.0) and median sFlt-1/PlGF ratio was 6.8 (IQR 4.5–9.7). In the nicotine exposure study group (*n* = 2278), median maternal sFlt-1 concentration was 1257.0 pg/ mL (IQR 937.8–1753.0), median PlGF was 193.0 pg/ mL (IQR 143.0–261.0) and median sFlt-1/PlGF ratio was 6.7 (4.4–9.7) (Table 2).

**Table 2:** Median maternal angiogenic biomarker levels for the Nicotine exposure study group (*n* = 2278)

Characteristics	N	%	sFlt-1, pg/mL		PlGF, pg/mL		sFlt-1/PlGF ratio	
			Median	IQR	Median	IQR	Median	IQR
*Total biomarker study group*	**2603**		1258.0	938.0–1754.0	192.0	142.0–260.0	6.8	4.5–9.7
*Nicotine exposure study group*	2278		1257.0	937.8–1753.0	193.0	143.0–261.0	6.7	4.4–9.7
*Snus*	2278							
Never	1762	77.3	1245.0	935.0–1729.3	195.0	144.0–263.0	6.6	4.4–9.6
Stopped before pregnancy	343	15.1	1334.0	971.0–1905.0	189.0	141.0–253.0	7.1	4.8–10.7
Stopped when recognizing pregnancy	160	7.0	1252.5	903.0–1752.8	183.5	143.3–265.0	7.1	4.6–9.4
Current	13	0.6	996.0	729.5–1392.5	166.0	93.5–204.5	6.6	5.2–8.9
*Smoking*	2217*							
Never	1762	79.4	1264.5	946.8–1754.3	192.0	143.0–261.3	6.8	4.5–9.8
Stopped before pregnancy	343	15.5	1238.5	915.3–1761.8	194.0	142.0–261.0	6.7	4.5–9.3
Stopped when recognizing pregnancy	97	4.4	1216.0	869.0–1687.0	189.0	142.0–247.5	6.7	4.6–9.9
Current	15	0.7	1146.0	820.0–1928.0	240.0	176.0–303.0	4.3	2.8–10.2

IQR, interquartile range; *n*, number; *P*, *p*-value; pg/mL, picograms per milliliter; PlGF, Placental Growth Factor; sFlt-1, Soluble Fms-like tyrosine kinase receptor 1; *, missing data

Descriptive data of PlGF and sFlt-1 as well as their ratio according to the different nicotine exposure categories and stratified by fetal sex are shown in Additional Table 1.

Women carrying a male fetus who reported snus use that ‘stopped before pregnancy’ (*n* = 167, 14.5%), ‘stopped when recognizing pregnancy’ (*n* = 76, 6.6%) or current snus use (*n* = 6, 0.5%) had low median PlGF [189.0 pg/L (IQR 136.0–245.0); 177.5 pg/L (IQR 134.5–249.8) and 176.5 pg/L (IQR 128.5–242.0) respectively] compared with women who ‘never used snus’ (*n* = 899, 78.3%) [205.0 pg/L (IQR 151.0–276.0)] (Additional file 1: Table S1). In women carrying a female fetus, PlGF was low among the six women reporting current snus use (Additional file 1: Table S1).

We found significant effects of fetal sex on sFlt-1, PlGF and the sFlt-1/ PlGF ratio. Multivariable linear regression analysis adjusted for gestational age, maternal age, prepregnancy BMI and parity showed that fetal sex was significantly associated with sFlt-1 [(*β* − 0.01, (95% CI − 0.04;(− 0.01)), *p* = 0.007), PlGF [(*β* 0.02, (95% CI 0.01;0.03), *p* = 0.003) and sFlt-1/PlGF ratio [(*β* − 0.04, (95% CI − 0.07; − 0.03), *p* < 0.001)] (Additional file 2: Table S2).

Current snus use was significantly associated with reduced maternal circulating PlGF levels in multivariable regression analyses [*β* − 0.12, (95% CI − 0.20; 0.00) compared to never use, *p* = 0.020] adjusting for gestational age, maternal age, prepregnancy BMI, parity and fetal sex, but no corresponding associations were observed for sFlt-1 or the sFlt-1/PlGF ratio (Table 3). Smoking was not significantly associated with any of the biomarker levels (Table 3).

**Table 3:** Effect of nicotine exposure on midpregnancy circulating angiogenic biomarkers (Nicotine exposure study group, *n* = 2278)

Nicotine exposure	N	%	Univariable								
			sFlt-1			PlGF			sFlt-1/ PlGF-ratio		
			*β*	95% CI	*P*	*β*	95% CI	*P*	*β*	**95% CI**	***P***
*Snus*	2090				0.091			0.023			0.072
Never (Ref.)	1622	77.6									
Stopped before pregnancy	313	15.0	0.02	− 0.01; 0.04		− 0.02	− 0.05;0.00		0.04	0.01;0.07	
Stopped when recognized pregnancy	143	6.8	− 0.01	− 0.05; 0.03		− 0.03	− 0.06;0.01		0.02	− 0.03;0.06	
Current	12	0.6	− 0.12	− 0.2;0.00		− 0.13	− 0.2;(− 0.01)		0.01	− 0.14;0.15	
*Smoke*	2200				0.530			0.360			0.282
Never (Ref.)	1702	77.3									
Stopped before pregnancy	388	17.6	− 0.01	− 0.03;0.01		− 0.00	− 0.03;0.02		− 0.01	− 0.04;0.02	
Stopped when recognized pregnancy	95	4.3	− 0.02	− 0.06;0.02		− 0.02	− 0.06;0.03		− 0.01	− 0.06;0.05	
Current	15	0.7	− 0.04	− 0.14;0.07		0.084	− 0.02;0.19		− 0.12	− 0.3;0.01	

Nicotine exposure	N	%	Multivariable											
			sFlt-1				PlGF				sFlt-1/ PlGF-ratio			
			*Β*	95% CI	*P*	*Pinteraction*	*Β*	95% CI	*P*	*Pinteraction*	*Β*	95% CI	*P*	*Pinteraction*
*Snus*	2090				0.157	0.116			**0.020**	**0.031**			0.271	0.233
Never (Ref.)	1622	77.6												
Stopped before pregnancy	313	15.0	0.00	− 0.02;0.03			− 0.03	− 0.05;0.00			0.03	0.00;0.06		
Stopped when recognized pregnancy	143	6.8	− 0.02	− 0.06;0.01			− 0.03	− 0.06;0.01			0.004	− 0.04;0.05		
Current	12	0.6	− 0.11	− 0.20;0.01			− 0.12	− 0.20;0.00			0.007	− 0.14;0.15		
*Smoke*	2200				0.530	0.552			0.248	0.104			0.228	0.361
Never (Ref.)	1702	77.3												
Stopped before pregnancy	388	17.6	− 0.01	− 0.03;0.01			− 0.00	− 0.03;0.02			− 0.008	− 0.04;0.02		
Stopped when recognized pregnancy	95	4.3	− 0.02	− 0.06;0.02			− 0.02	− 0.06;0.03			− 0.005	− 0.06;0.05		
Current	15	0.7	− 0.03	− 0.14;0.07			0.1	− 0.06;0.20			− 0.13	− 0.3;(− 0.003)		

Uni- and multivariable linear regression analyses on log transformed biomarker concentrations. Multivariable linear regression analyses are adjusted for fetal sex, gestational age, maternal age, prepregnancy BMI and parity (*N* = 2200). Global *p*-values are shown

B, beta coefficient; CI, confidence interval, *N*, number; *P*, global *p*-value; Pinteraction, interaction term between fetal sex and snus use on biomarker levels; PlGF, Placental Growth Factor; Ref., reference group; sFlt-1, Soluble Fms-like tyrosine kinase receptor 1

**Fig. 2:**
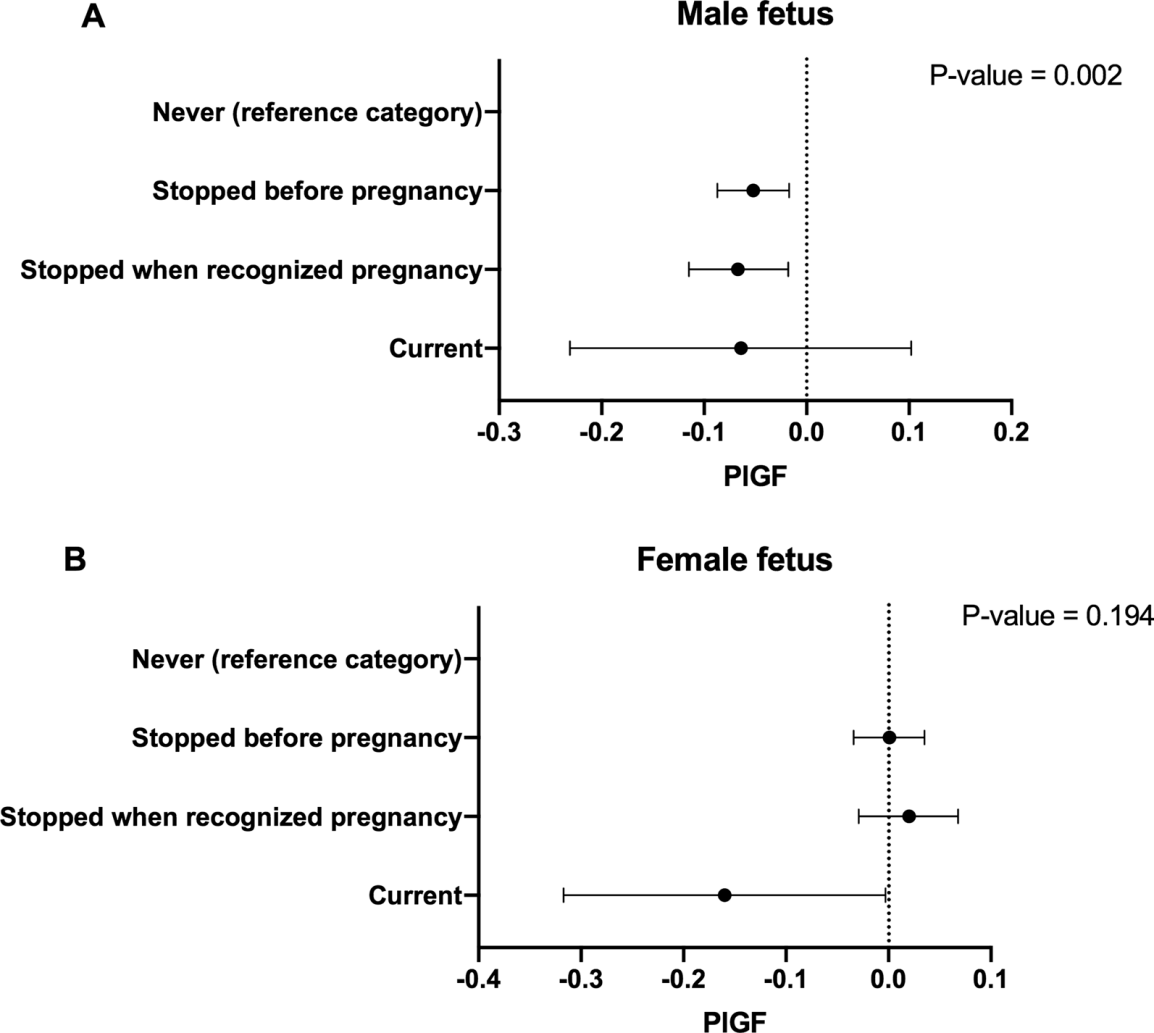
Effect of snus use on circulating midpregnancy maternal angiogenic biomarker levels by fetal sex. **A** Pregnancies with a male fetus (*n* = 1201), B: pregnancies with a female fetus (*n* = 1066). PlGF, Placental Growth Factor. Multivaribale linear regression analyses on log transformed biomarker levels

We found a significant interaction between fetal sex and snus exposure for PIGF (*p* = 0.031) but not for the other biomarkers or smoking (Table 3). While snus use was significantly associated with PIGF in male fetus pregnancies [‘stopped before pregnancy’: *β* − 0.05 (95% CI − 0.09 to (− 0.02)), ‘stopped when recognizing pregnancy’: *β* − 0.07 (95% CI − 0.12 to (− 0.02)); ‘current’: *β* − 0.06 (95% CI − 0.23 to 0.10)] compared to women who had never used snus, *p* = 0.002) (Fig. 2A, B), there was no association between snus use and PIGF in female pregnancies (*p* = 0.194; Additional file 3: Table S3).

Since there was no significant interaction between fetal sex and smoking, stratification by fetal sex with further biomarker level analyses was not done for this group. Removing the second pregnancy of the four women contributing with two separate pregnancies did not alter the results nor conclusions (data not shown).

## Discussion

To the best of our knowledge, our study is the first to report the effect of snus before or during pregnancy on midpregnancy placenta-associated biomarkers. Snus use in pregnancy was associated with lower PlGF levels, but not with sFlt-1 or the sFlt-1/PlGF ratio, while no significant associations were found for smoking. Our study of more than 2600 women is one of the largest to confirm that maternal circulating placenta-associated angiogenic biomarkers differ according to fetal sex. We found significantly lower antiangiogenic pattern in male fetus pregnancies, i.e., lower sFlt-1 concentration, higher PlGF and lower sFlt-1/PlGF ratio, which was confirmed after adjustment for preselected potential confounders in multivariable analyses. We found low PlGF levels in male fetus pregnancies exposed to nicotine in the form of snus before or during early pregnancy, but not in female fetus pregnancies, indicating a sex dimorphic effect of snus on maternal circulating placenta-associated angiogenic proteins. Since maternal circulating proangiogenic PlGF and antiangiogenic sFlt-1 may be seen as “placenta health markers” during pregnancy [1], our finding of fetal sex-specific differences in response to nicotine exposure is of importance. In our study sFlt-1 and PlGF levels as well as sFlt-1/PlGF ratio were similar, as reported in a previously published study by Verlohren et al. [22] that included 157 women at 15–19 GW and 217 women at 20–23 GW. Our finding of a sexual dimorphism in maternal circulating angiogenic factor levels with significantly higher antiangiogenic pattern in female compared to male fetus pregnancies is in line with previous reports from first trimester [16], as well as throughout pregnancy [17].

The lack of significant associations between nicotine exposure in the form of snus in pregnancy and angiogenic factor levels in adjusted analyses may be due to the low numbers of current snus users since most women stopped in early first trimester [9]. Mijal et al. suggested that since changes in circulating angiogenic marker levels are more pronounced in late pregnancy, an effect of smoking may have a greater impact towards term [23]. This same line of argumentation may be true for snus use.

The angiogenic placenta-associated biomarkers have a likely effect on early placentation processes, including endometrial and trophoblast function, feto-maternal immune interactions and uteroplacental spiral artery remodeling [24]. Maternal age, prepregnancy BMI, parity, gestational age and fetal sex were chosen as preselected potential confounders in the multivariable analyses due to their impact on placental function.

A strength of the present study is that our well-described, relatively homogenous pregnancy population allowed an evaluation of both the impact of fetal sex, nicotine use and maternal characteristics on placenta health-related biomarkers. Another strength is, that our study population was recruited from a relatively small gestational age window, compared to an earlier study [23]. In addition, albeit including 3 study sites, we had less dissemination of study sites compared to Mijal et al., at the same time reaching a similar number of study participants [23]. We regard this a strength, since adherence to a study protocol is easier surveyed when fewer study sites are included.

The generalizability of our study is restricted by low ethnic diversity, similar to Andersen et al. had in their Danish study with only 3.5% of the women originating from non-Western countries [17]. The high mean maternal age and educational level in our study may potentially cause an underestimation of nicotine use. However, snus use in pregnancy has previously been shown to be inversely associated with age [9], and younger women are more likely to use snus prior to pregnancy [25]. Self-reports of nicotine use and the risk of recall bias may result in an underestimation of use. We have no objective measure of nicotine use, i.e. blood cotinine levels were not assessed. However, studies comparable to ours have found a high association between self-reported data and nicotine exposure by blood cotinine in pregnant women, indicating that self-reporting data are valid [26, 27]. We acknowledge that in our study, cessation before pregnancy could span from months to years. Also, there may have been some prior smokers in all snus categories, potentially affecting biomarker levels.

## Perspectives and significance

Our finding of a significant interaction between fetal sex and snus exposure on placental function is novel, and suggests a possible sustained long term “antiangiogenic” effect of snus in male fetus pregnancies. This finding is in line with other aspects of fetal sex-specific adaptation to environmental factors [28, 29], with male fetuses being more vulnerable to exogenous insults than female fetuses. Sex-specific differences in placental health should be accounted for in future biomarker studies.

## Conclusions

Our study suggests that exposure to snus before and in early pregnancy has a sex-dimorphic effect on the midpregnancy maternal circulating pro- and antiangiogenic protein profiles. The lower proangiogenic PlGF level in women carrying a male fetus supports the notion that male fetuses are more vulnerable to exogenous insults than female fetuses from early stages of pregnancy. Among Scandinavian women in reproductive age the use of alternative nicotine products, such as snus is increasing. Since snus use is a modifiable risk factor for adverse pregnancy outcomes, further research should investigate whether our observed fetal sex specific differences in placental health at midpregnancy translate into differences in pregnancy and delivery outcome.

## Supplementary Information

**Additional file 1: Table S1.** Nicotine exposure by fetal sex and midpregnancy maternal biomarker concentrations for the Nicotine exposure study group (*n* = 2278).

**Additional file 2: Table S2.** Effect of fetal sex on midpregnancy angiogenic biomarkers (Nicotine exposure group, *n* = 2278).

**Additional file 3: Table S3.** Effect of nicotine exposure on midpregnancy PlGF, stratified by fetal sex.

## Data Availability

The minimal data set that support the findings of this study are available upon reasonable requests from to access underlying our study can be sent to Ingvil Krarup Sørbye, MD, PhD, Head of Department of Research, Division of Obstetrics and Gynaecology, Oslo University Hospital, Postboks 4956, Nydalen, 0424 Oslo, Norway, e-mail isorbye@ous-hf.no, but access to the full data set is restricted due to ongoing clinical follow-up, the sensitive nature of the data collected for this study, and the restriction due to patient informed consents (and thereby ethical body approval).
