# Supplementary material for: The effect of nicotine-containing products and fetal sex on placenta-associated circulating midpregnancy biomarkers
Source: Biol Sex Differ. 2022 Jul 15;13:39. doi: 10.1186/s13293-022-00443-1 (PMC9284818; doi:10.1186/s13293-022-00443-1)
Supplement: Supplementary file 1 — Additional file 1: Table S1. Nicotine exposure by fetal sex and midpregnancy maternal biomarker concentrations for the Nicotine exposure study group (n = 2278). [file 13293_2022_443_MOESM1_ESM.docx]

Additional Table 1. Nicotine exposure by fetal sex and midpregnancy maternal biomarker concentrations for the Nicotine exposure study group (n=2278)

| **Nicotine exposure** |  |  | **sFlt1 (pg/mL)** | | **PlGF (pg/ mL)** | | **sFlt1/PlGF-ratio** | |
| --- | --- | --- | --- | --- | --- | --- | --- | --- |
|  | **N** | **%** | **Median** | **IQR** | **Median** | **IQR** | **Median** | **IQR** |
| **Snus, male fetus** | 1148 |  |  |  |  |  |  |  |
| Never | 899 | 78.3 | 1234.0 | 920.0-1700.0 | 205.0 | 151.0-276.0 | 6.2 | 4.2-8.9 |
| Stopped before pregnancy | 167 | 14.5 | 1236.0 | 917.0-752.0 | 189.0 | 136.0-245.0 | 6.4 | 4.6-9.9 |
| Stopped when recognizing pregnancy | 76 | 6.6 | 1240.0 | 913.5-1632.3 | 177.5 | 134.5-249.8 | 7.1 | 4.8-9.5 |
| Current | 6 | 0.5 | 961.0 | 710.0-1107.0 | 176.5 | 128.5-242.0 | 5.4 | 3.0-7.1 |
| **Snus, female fetus** | 1007 |  |  |  |  |  |  |  |
| Never | 772 | 76.6 | 1259.0 | 958.0-1779.0 | 183.0 | 139.3-249.8 | 7.1 | 4.8-10.3 |
| Stopped before pregnancy | 157 | 15.6 | 1486.0 | 1288.5-2057.5 | 185.0 | 141.0-260.0 | 7.6 | 5.4-11.1 |
| Stopped when recognizing pregnancy | 72 | 7.1 | 1332.5 | 882.5-1775.0 | 183.5 | 143.3-289.0 | 7.1 | 4.7-9.5 |
| Current | 6 | 0.6 | 1209.0 | 622.3-1961.0 | 104.0 | 91.0-213.8 | 8.0 | 5.7-13.1 |
| **Smoke, male fetus** | 1201 |  |  |  |  |  |  |  |
| Never | 929 | 77.3 | 1240.0 | 924.0-1706.0 | 201.0 | 148.0-274.0 | 6.2 | 4.2-9.0 |
| Stopped before pregnancy | 219 | 18.2 | 1206.0 | 910.0-1650.0 | 195.0 | 142.0-260.0 | 6.3 | 4.6-8.9 |
| Stopped when recognizing pregnancy | 49 | 4.1 | 1130.0 | 828.5-1645.0 | 189.0 | 137.0-33.0 | 6.6 | 4.6-8.2 |
| Current | 4 | 0.3 | 1537.0 | 907.5-1964.0 | 240.0 | 194.8-588.3 | 4.1 | 3.1-8.7 |
| **Smoke, female fetus** | 1066 |  |  |  |  |  |  |  |
| Never | 824 | 77.3 | 1287.0 | 982.8-1828.0 | 182.5 | 139.0-248.5 | 7.2 | 4.9-10.4 |
| Stopped before pregnancy | 183 | 17.2 | 1309.0 | 932.0-1844.0 | 190.0 | 141.0-269.0 | 7.2 | 4.5-10.1 |
| Stopped when recognizing pregnancy | 48 | 4.5 | 1270.0 | 930.8-1838.8 | 181.5 | 146.0-259.0 | 7.0 | 4.8-10.7 |
| Current | 11 | 1.0 | 1095.0 | 766.0-1485.0 | 240.0 | 165.0-303.0 | 5.5 | 2.8-10.4 |
| IQR=interquartile range; N=number; pg/mL= picograms per milliliter; PlGF= Placental Growth Factor; sFlt-1=Soluble Fms-like tyrosine kinase receptor 1. | | | | | | | | |
