# Supplementary material for: The effect of nicotine-containing products and fetal sex on placenta-associated circulating midpregnancy biomarkers
Source: Biol Sex Differ. 2022 Jul 15;13:39. doi: 10.1186/s13293-022-00443-1 (PMC9284818; doi:10.1186/s13293-022-00443-1)
Supplement: Supplementary file 3 — Additional file 3: Table S3. Effect of nicotine exposure on midpregnancy PlGF, stratified by fetal sex. [file 13293_2022_443_MOESM3_ESM.docx]

Additional Table 3. Effect of nicotine exposure on midpregnancy PlGF, stratified by fetal sex

|  |  |  |  | **Male** |  |  |  |  | **Female** |  |
| --- | --- | --- | --- | --- | --- | --- | --- | --- | --- | --- |
|  | **N** | % | **b** | **95% CI** | ***P*** | **N** | % | **b** | **95% CI** | ***P*** |
| **Snus** | 1127 |  |  |  | **0.002** | 963 |  |  |  | 0.194 |
| Never (Ref.) | 883 | 78.3 |  |  |  | 739 | 76.7 |  |  |  |
| Stopped before pregnancy | 163 | 14.5 | -0.052 | -0.087; -0.017 |  | 150 | 15.6 | 0.001 | -0.034; 0.035 |  |
| Stopped when recognized pregnancy | 75 | 6.7 | -0.067 | -0.115: -0.018 |  | 68 | 7.1 | 0.020 | -0.029;0.068 |  |
| Current | 6 | 0.5 | -0.064 | -0.231; 0.102 |  | 6 | 0.6 | -0.160 | -0.317; -0.003 |  |
| B=beta coefficient; CI=confidence interval, N=number; *P*= global *p*-value; PlGF= Placental Growth Factor; Ref.=reference group | | | | | | | | | | |
